# Angiotensin-(1-7) Improves Integrated Cardiometabolic Function in Aged Mice

**DOI:** 10.3390/ijms21145131

**Published:** 2020-07-20

**Authors:** Amanda J. Miller, Sarah S. Bingaman, Darren Mehay, Daniela Medina, Amy C. Arnold

**Affiliations:** Department of Neural and Behavioral Sciences, Pennsylvania State University College of Medicine, Hershey, PA 17033, USA; aross1@pennstatehealth.psu.edu (A.J.M.); ssimmonds@pennstatehealth.psu.edu (S.S.B.); dmehay@pennstatehealth.psu.edu (D.M.); dmedina@pennstatehealth.psu.edu (D.M.)

**Keywords:** renin–angiotensin system, aging, blood pressure, sympathetic, autonomic, insulin sensitivity

## Abstract

Angiotensin (Ang)-(1-7) is a beneficial renin–angiotensin system (RAS) hormone that elicits protective cardiometabolic effects in young animal models of hypertension, obesity, and metabolic syndrome. The impact of Ang-(1-7) on cardiovascular and metabolic outcomes during aging, however, remains unexplored. This study tested the hypothesis that Ang-(1-7) attenuates age-related elevations in blood pressure and insulin resistance in mice. Young adult (two-month-old) and aged (16-month-old) male C57BL/6J mice received Ang-(1-7) (400 ng/kg/min) or saline for six-weeks via a subcutaneous osmotic mini-pump. Arterial blood pressure and metabolic function indices (body composition, insulin sensitivity, and glucose tolerance) were measured at the end of treatment. Adipose and cardiac tissue masses and cardiac RAS, sympathetic and inflammatory marker gene expression were also measured. We found that chronic Ang-(1-7) treatment decreased systolic and mean blood pressure, with a similar trend for diastolic blood pressure. Ang-(1-7) also improved insulin sensitivity in aged mice to levels in young mice, without effects on glucose tolerance or body composition. The blood pressure–lowering effects of Ang-(1-7) in aged mice were associated with reduced sympathetic outflow to the heart. These findings suggest Ang-(1-7) may provide a novel pharmacological target to improve age-related cardiometabolic risk.

## 1. Introduction

Cardiovascular disease remains the leading cause of death in individuals greater than 65 years of age [1]. Aging is associated with increased arterial stiffness, changes in cardiac structure and function, reduced parasympathetic tone, and elevated sympathetic outflow to the heart and other cardiovascular organs. These age-related manifestations predispose to increased risk for developing cardiovascular diseases including systolic hypertension, stroke, myocardial infarction, and heart failure [1]. Aging is also associated with weight gain and metabolic dysfunction (e.g., insulin resistance, glucose intolerance, and type 2 diabetes), which exacerbates the progression of cardiovascular disease to increase morbidity and mortality [2]. Given the increasing aging population worldwide, it is important to understand pathophysiological mechanisms contributing to age-related impairments in cardiovascular and metabolic functions. 

The renin–angiotensin system (RAS) may provide an important hormonal link connecting cardiovascular and metabolic derangements in aging. Angiotensin (Ang) II is well recognized to bind type 1 receptors (AT1R) to increase blood pressure via neural, renal, vascular, and cardiac mechanisms [3,4]. Ang II is implicated in numerous cardiovascular diseases, with more recent evidence showing a role for elevated levels of this hormone in insulin resistance, obesity, and type 2 diabetes [5,6]. While plasma renin activity and responsiveness and Ang II levels decline during aging, AT1R density is increased to enhance sensitivity to Ang II at the local tissue level [7,8]. Consistent with this, therapies blocking Ang II activity such as angiotensin-converting enzyme (ACE) inhibitors and angiotensin receptor blockers (ARBs) effectively lower blood pressure, reduce risk for diabetes, prevent deficits in cardiovascular and metabolic functions and increase lifespan in aged animals and clinical populations [7,9,10,11]. Of interest, ACE inhibitors and ARBs increase levels of the protective hormone Ang-(1-7), which our group and others have shown contributes to the beneficial cardiometabolic effects of these therapies in animal models of cardiovascular diseases, obesity, and diabetes [12,13,14,15]. Importantly, ACE inhibitors are limited in up to 11% of patients by cough due to production of kinins, and many patients are unresponsive to these therapies in terms of blood pressure control [16]. Direct targeting of Ang-(1-7) may provide benefit beyond ACE inhibitors and ARBs by improving integrated cardiometabolic function while avoiding limiting side effects of these therapies. 

Ang-(1-7) is a more recently discovered RAS hormone that binds Mas receptors to oppose the deleterious cardiovascular and metabolic actions of Ang II [17,18]. In young adult rodent models of obesity and metabolic syndrome, circulating Ang-(1-7) levels are reduced, and chronic restoration of this hormone improves blood pressure control as well as measures of metabolic function such as body mass, hyperinsulinemia, insulin sensitivity, glucose tolerance, and lipid metabolism [19,20,21,22,23]. Ang-(1-7) also attenuates cardiac hypertrophy, fibrosis, and post-infarct remodeling in young adult rodent models of hypertension and obesity [19,24]. Emerging evidence suggests that aging is also associated with decreased levels of Ang-(1-7) in the circulation as well as reduced vasodilatory responses to exogenous Ang-(1-7) administration in isolated aortic vessels [25,26,27]. To our knowledge, there are no studies evaluating the importance of Ang-(1-7) treatment in vivo to integrated cardiovascular and metabolic outcomes during aging. In this study, we tested the hypothesis that Ang-(1-7) attenuates age-related elevations in blood pressure and insulin resistance. We also examined if any blood pressure lowering effects of Ang-(1-7) in aged mice were associated with changes in indices of cardiac autonomic tone.

## 2. Results

### 2.1. Ang-(1-7) Does Not Affect Body Weight or Composition in Aged Mice

Body mass and adipose and cardiac tissue weights are shown in Table 1. Aged mice had a higher body mass compared to young mice. In aged mice, adiposity and fluid mass percentages were also higher, and percent lean mass was lower. Chronic Ang-(1-7) treatment did not affect body mass or body composition in aged or young mice. The weights of epididymal and inguinal white adipose, brown adipose, and cardiac tissue were higher in aged, compared to young mice, but were not significantly affected by Ang-(1-7) treatment.

### 2.2. Ang-(1-7) Improves Insulin Sensitivity in Aged Mice

**Figure 1:**
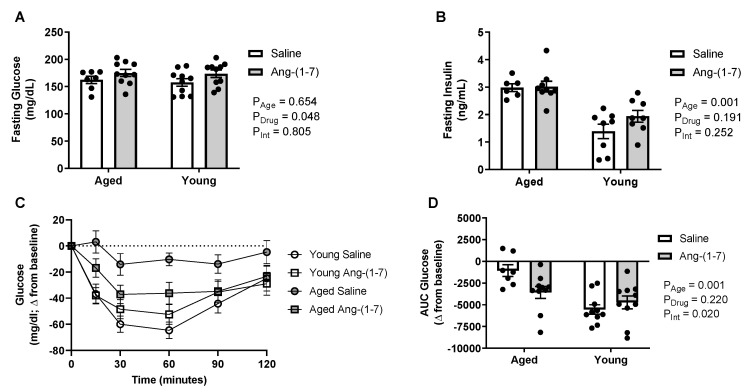
Angiotensin (Ang)-(1-7) improved insulin sensitivity in aged mice (*n* = 8–10/group). (**A**), Bar charts showing there was no effect of aging on circulating glucose levels measured after a 4-h fasting period. The Ang-(1-7) treatment mildly elevated glucose compared with saline in young adult and aged mice. (**B**), Bar charts showing aged mice had elevated circulating insulin levels measured after a 4-h fasting period, which was not reversed by Ang-(1-7) treatment. (**C**), Raw data curve showing changes in blood glucose levels from baseline over a 120-min period following exogenous insulin administration in young adult and aged mice. Dotted line represents baseline. (**D**), Glucose data summarized as an area under the curve (AUC), with a more negative number indicating a greater drop in glucose in response to insulin or increased insulin sensitivity. Aged mice had a significant reduction in insulin sensitivity, which was improved following chronic Ang-(1-7) treatment to a similar level to that observed in young adult mice. Data are mean ± SEM and were analyzed by two-way ANOVA for main effects of age (P_Age_) and Ang-(1-7) versus saline treatment (P_Drug_) as well as their interaction (P_Int_), with Tukey post-hoc pairwise comparisons with correction for multiple comparisons.

Aged mice developed a modest hyperinsulinemia, in the absence of changes in fasting blood glucose levels following a four-hour fasting period (Figure 1A,B), suggesting insulin resistance. There was a significant main effect of Ang-(1-7) to increase glucose (on average 12–16 mg/dL), without affecting insulin. The decrease in blood glucose in response to exogenous insulin during the insulin tolerance test (ITT) is shown in Figure 1C. The area under the curve (AUC) was calculated to summarize changes in glucose in response to insulin, with a more negative value indicating higher insulin sensitivity (Figure 1D). Insulin sensitivity was significantly lower in aged mice compared to in young mice (Figure 1D). While no main effect of Ang-(1-7) treatment was evident, there was a significant interaction detected, with Ang-(1-7) improving insulin sensitivity in aged mice to a similar level to that observed in young mice.

### 2.3. Glucose Tolerance Is Not Affected by Either Aging or Ang-(1-7) Treatment

**Figure 2:**
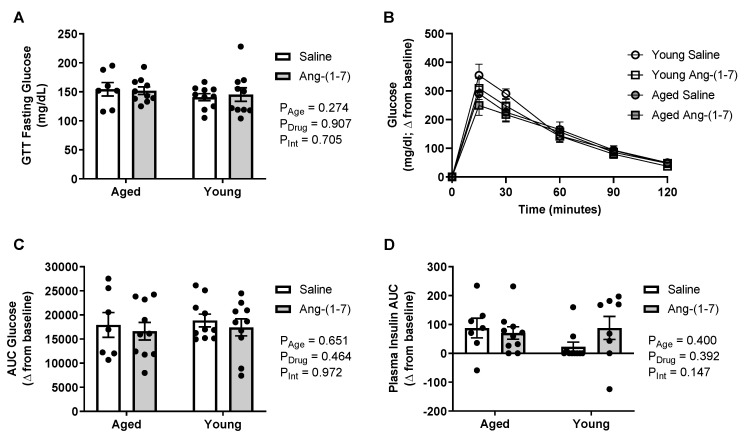
Glucose tolerance was not affected by aging or chronic angiotensin (Ang)-(1-7) treatment (*n* = 8–10/group). (**A**), Bar charts showing there was no impact of aging or Ang-(1-7) treatment on circulating glucose levels measured after an overnight fasting period. (**B**), Raw data curve showing changes in blood glucose levels from baseline over a 120-min period following exogenous dextrose administration in young adult and aged mice. (**C**), Glucose data summarized as an AUC, with a more positive number indicating higher levels of glucose remaining in the blood over time or glucose intolerance. There was no effect of aging or Ang-(1-7) treatment on glucose tolerance. (**D**), Insulin data summarized as an AUC, with a more positive number indicating increased insulin secretion. There was no effect of aging or Ang-(1-7) treatment on glucose-stimulated endogenous insulin secretion. Data are mean ± SEM and were analyzed by two-way ANOVA for main effects of age (*P*_Age_) and Ang-(1-7) versus saline treatment (*P*_Drug_) as well as their interaction (P_Int_), with Tukey post-hoc pairwise comparisons with correction for multiple comparisons.

There was no impact of either aging or Ang-(1-7) treatment on circulating glucose levels when measured after an overnight fast (Figure 2A). The increase in blood glucose in response to exogenous dextrose administration during the glucose tolerance test (GTT) is shown in Figure 2, with a more positive AUC value indicating glucose intolerance. There were no main effects of age or Ang-(1-7) treatment on glucose tolerance (Figure 2B,C) as well as no interaction. During the GTT, the AUC for change in plasma insulin in response to dextrose was also measured to examine for potential changes in glucose-stimulated endogenous insulin secretion, with a higher AUC indicating increased insulin secretion. Similar to the findings for glucose tolerance, there was no effect of either age or Ang-(1-7) treatment on insulin secretion (Figure 2D).

### 2.4. Ang-(1-7) Decreases Systolic and Mean Blood Pressure in Aged Mice

**Figure 3:**
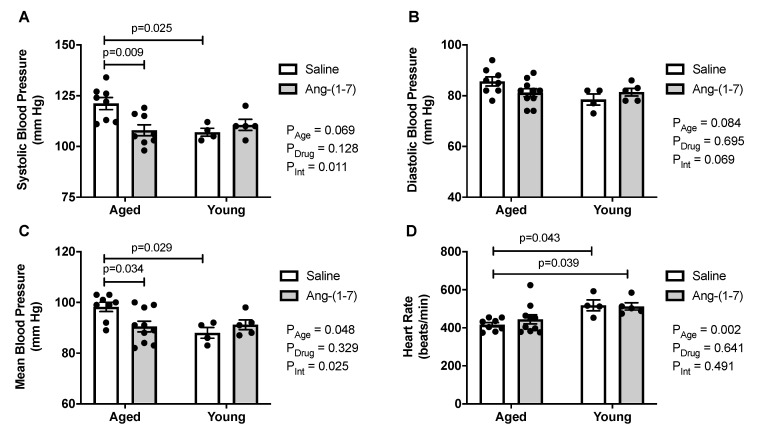
Angiotensin (Ang)-(1-7) decreased blood pressure in aged mice (*n* = 4–10/group). (**A**), Bar charts showing aged mice exhibited an increase in systolic blood pressure, which was reduced by Ang-(1-7) to levels seen in young adult mice. (**B**), Bar charts showing there was no effect of either age or Ang-(1-7) treatment on diastolic blood pressure. (**C**), Bar charts showing aged mice had an increase in mean blood pressure, which was reduced by Ang-(1-7) to levels seen in young adult mice. (**D**), Bar charts showing aged mice had a lower heart rate compared with young mice, which was not impacted by Ang-(1-7) treatment. Data are mean ± SEM and were analyzed by two-way ANOVA for main effects of age (*P*_Age_) and Ang-(1-7) versus saline treatment (*P*_Drug_) as well as their interaction (*P*_Int_), with Tukey post-hoc pairwise comparisons with correction for multiple comparisons.

Aged mice exhibited higher systolic and mean blood pressures compared with young mice, with a similar trend for diastolic blood pressure (Figure 3A,C). Ang-(1-7) reduced systolic and mean blood pressure in aged mice, with no effect on diastolic blood pressure. Heart rate was reduced in aged mice, compared to in young mice (Figure 3D), but was not affected by Ang-(1-7) treatment.

### 2.5. Ang-(1-7) Decreases Measures of Cardiac Sympathetic Tone in Aged Mice

**Figure 4:**
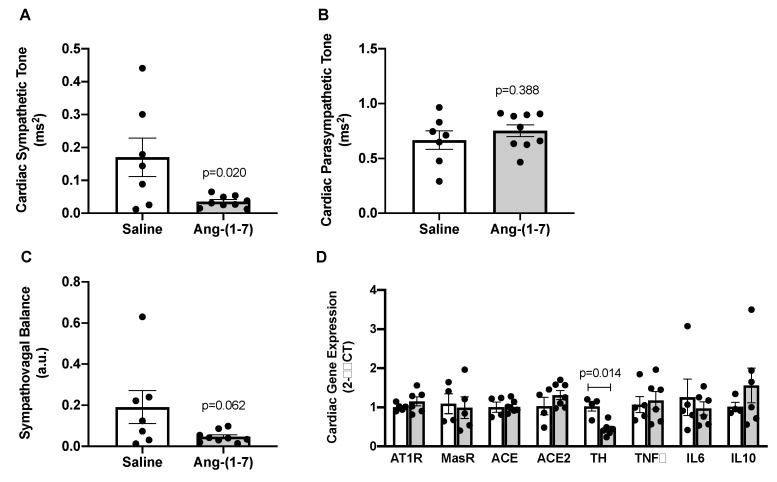
Angiotensin (Ang)-(1-7) decreased measures of cardiac sympathetic tone in aged mice. (**A**–**C**), Indices of cardiac sympathetic and parasympathetic tone measured by spectral analysis of low-frequency and high-frequency components of heart rate variability, respectively (*n* = 7–9/group). Ang-(1-7) reduced cardiac sympathetic tone, with no effect on parasympathetic tone, in aged mice. This resulted in a trend for Ang-(1-7) to improve overall cardiac sympathovagal balance in aged mice. (**D**), Gene expression measured in hearts from saline- versus Ang-(1-7)-treated aged mice using quantitative real-time PCR methods (*n* = 4–6/group). Ang-(1-7) reduced cardiac mRNA of tyrosine hydroxylase (TH), the rate-limiting enzyme for norepinephrine synthesis, in the heart of aged mice. There was no effect of Ang-(1-7) on cardiac mRNA for markers of the renin–angiotensin system (AT1R, Ang II AT1 receptor; MasR, Ang-(1-7) Mas receptor; ACE, angiotensin-converting enzyme; ACE2, angiotensin-converting enzyme 2) or inflammatory markers (TNFα, tumor necrosis factor α; IL6, interleukin 6; IL10, interleukin 10). Data are mean ± SEM and were analyzed by an unpaired *t*-test.

To determine potential mechanisms underlying blood pressure-lowering effects of Ang-(1-7) in aged mice, cardiac autonomic tone was assessed via spectral analysis in a subset of aged mice treated with saline versus Ang-(1-7). We found that Ang-(1-7) significantly decreased cardiac sympathetic tone in aged mice (Figure 4A), with no effect on cardiac parasympathetic tone (Figure 4B). This resulted in a trend towards improved sympathovagal balance in Ang-(1-7)-treated aged mice (Figure 4C). Cardiac gene expression of tyrosine hydroxylase (Th), a marker of sympathetic neuronal activity, was significantly reduced by Ang-(1-7) treatment in aged mice, further suggesting reduced cardiac sympathetic tone (Figure 4D). Cardiac gene expression of RAS (AT1R, Mas receptor, ACE, and angiotensin-converting enzyme 2 (ACE2)) and inflammatory (tumor necrosis factor α (TNFα), interleukin 6 (IL6), and interleukin 10 (IL10)) markers were unaffected by Ang-(1-7) treatment in aged mice (Figure 4D).

## 3. Discussion

Despite numerous studies showing protective cardiometabolic effects in young animals [6,17,18], there are few studies examining Ang-(1-7) actions in aged animals. The goal of this study was to determine whether chronic Ang-(1-7) treatment improved age-related cardiovascular and metabolic deterioration. The main findings in aged mice are as follows: (1) a cardiometabolic phenotype similar to aging in clinical populations was observed including the increased body mass and adiposity, hyperinsulinemia, insulin resistance, cardiac hypertrophy, elevated blood pressure, and reduced heart rate; (2) Ang-(1-7) infusion for six weeks improved measures of integrated cardiometabolic function, specifically systolic and mean blood pressure and insulin sensitivity; (3) Ang-(1-7)-mediated improvements in blood pressure and insulin sensitivity were independent of changes in body mass, body composition, and cardiac hypertrophy; and (4) blood pressure-lowering effects of Ang-(1-7) were associated with reduced measures of sympathetic tone to the heart, in the absence of changes in local RAS and inflammatory markers. These overall data indicated that Ang-(1-7) may provide a novel target to reverse age-related cardiovascular and metabolic complications. To our knowledge, this is the first report describing effects of Ang-(1-7) on cardiometabolic outcomes in aged animal models. Therefore, while descriptive in nature, this study adds to the very limited literature on Ang-(1-7) in aging and provides a rationale for further research to identify precise mechanisms underlying these beneficial effects. 

Healthy aging is known to alter activity and responsiveness of the RAS. Plasma renin activity and responsiveness and aldosterone and Ang II levels are decreased in aged animals and clinical populations [7,8]. Ang II elicits exaggerated responses in aged animals, however, due to the increased tissue production of Ang II and the expression of AT1R [7,26,28,29]. In addition to increased Ang II actions, emerging studies suggest that circulating Ang-(1-7) levels are reduced with aging in human subjects, expression of Mas receptors and ACE2 are decreased in aorta of aged mice, and there is loss of vasodilatory responses to Ang-(1-7) in aorta of aged female mice [25,26,27]. This increased Ang II activity combined with decreased protective effects of Ang-(1-7) pathways can contribute to an increase in reactive oxygen species, mitochondrial dysfunction, and end-organ damage in aging [7,30]. The mechanisms underlying this potential shift in the Ang II to Ang-(1-7) balance in aging have not been investigated but may include changes in formation and degradation enzymatic pathways, changes in receptor density and related signaling pathways, as well as altered release of angiotensin peptides from local tissues into the circulation. A limitation of our study is we did not examine for changes in these potential circulating or tissue RAS mechanisms in response to either aging or chronic Ang-(1-7) infusion, and this will be an important future direction. 

Aging is an independent risk factor for systolic hypertension, which is largely attributed to increased arterial stiffness and higher sympathetic nervous system activity [31,32]. Diastolic blood pressure and heart rate decrease with age in humans [32,33]. In male mice, systolic and mean blood pressures increase with age, but diastolic blood pressure and heart rate remain stable throughout the lifespan [34]. Similarly, in our study, systolic and mean blood pressures increased with age and were selectively reduced by Ang-(1-7) treatment. We also observed a decrease in heart rate with aging that was not affected by Ang-(1-7), while diastolic blood pressure was not altered by either age or Ang-(1-7) treatment. These findings are consistent with previous studies, in which Ang-(1-7) decreased mean blood pressure in aged rats [35] and in young adult rodent models of metabolic syndrome and hypertension [36,37,38]. 

The blood pressure-lowering effects of Ang-(1-7) in aged mice were associated with decreased cardiac sympathetic tone measured by spectral analysis of heart rate variability. We also observed Ang-(1-7) decreased gene expression of Th, a rate-limiting enzyme for norepinephrine synthesis, in aged hearts. This suggests Ang-(1-7) lowers blood pressure via cardiac sympathoinhibitory mechanisms, a finding consistent with a previous study in metabolic syndrome rats [36]. We did not observe effects on cardiac parasympathetic tone in aged mice, which contrasts previous studies showing improvements in parasympathetic measures such as heart rate variability and arterial baroreflex sensitivity with Ang-(1-7) in younger animals [17]. A limitation is that we did not have cardiac samples from the young mice to determine if aging itself altered markers of sympathovagal balance prior to the Ang-(1-7) treatment. Alternate mechanisms that could contribute to depressor effects of Ang-(1-7) in aging include the restoration of attenuated vasodilatory responses [25,26], reductions in arterial stiffness, and the inhibition of local Ang II-ACE-AT1R pathways. Our data suggest that blood pressure-lowering effects of Ang-(1-7) are independent of changes in local RAS signaling and inflammatory pathways in the heart, although we did not assess for changes in Ang-(1-7) or Ang II peptide content. 

Aging is associated with increased cardiac mass, indicating left ventricular hypertrophy, which can occur even in the absence of hypertension [33]. While we observed age-related increases in cardiac mass in this study, it was not improved by Ang-(1-7) treatment. This contrasts previous studies, in which chronic Ang-(1-7) treatment improved cardiac function, reduced cardiac fibrosis and prevented cardiac remodeling following ischemia in young rodent models of hypertension, obesity, and metabolic syndrome [19,24,37,38]. In addition, ARBs reverse cardiac remodeling in aged spontaneously hypertensive rats, although this could reflect effects of reduced Ang II versus increased Ang-(1-7) [39]. It is also possible that six weeks of Ang1-7 treatment is not sufficient to reverse age-related impairments in cardiac structure and that more long-term infusions are needed. In addition, the aged mice in this study exhibited mild cardiac hypertrophy compared with the other models, which may not be improved with Ang-(1-7).

Aging is one of the strongest known risk factors for developing insulin resistance and type 2 diabetes [40]. Insulin resistance in aging is partially attributed to skeletal muscle aging leading to decreased expression of skeletal muscle glucose transporter type 4 (Glut4) and mitochondrial dysfunction [41]. In the current study, we observed that aged mice were insulin-resistant, which was reversed by chronic Ang-(1-7) treatment. Insulin-sensitizing effects of Ang-(1-7) have been demonstrated in lean rats and in rodent models of obesity, diabetes, and metabolic syndrome [20,21,22,42,43,44]. The mechanisms reported for insulin sensitization include improved intracellular insulin signaling and increased glucose uptake in peripheral tissues. We previously showed that, in obese male mice, Ang-(1-7) improves skeletal muscle insulin sensitivity by increasing Glut4 in the sarcolemma [21]. We suspect that Ang-(1-7) improves insulin sensitivity via a similar mechanism in aged mice, but further studies using hyperinsulinemic-euglycemic insulin clamps will be needed to confirm this. While not examined in this study, Ang-(1-7) could also induce changes in local RAS components in insulin-sensitive tissues such as skeletal muscle and adipose to contribute to improving insulin sensitivity in aged mice. Of interest, a recent study showed that Ang-(1-7) restores muscle strength in aged mice without changes in Ang II content in skeletal muscle or ACE2, Mas receptor, or AT1R expression in skeletal muscle and epididymal adipose tissue [45]. This finding, combined with our data showing lack of changes in RAS receptor and enzyme gene expression the heart, may suggest that beneficial physiological effects of Ang-(1-7) in aging are independent of local RAS signaling pathways. Further studies are needed, however, to confirm these findings. 

While aging is also known to promote glucose intolerance, aged mice in this study were not glucose–intolerant and thus there was no effect of Ang-(1-7) treatment. This is consistent with our previous studying showing that Ang-(1-7) does not improve glucose tolerance in obese male mice [43]. This contrasts other studies in male rat models of hypertension and metabolic syndrome, however, showing improved glucose tolerance with chronic Ang-(1-7) administration [20,22,36,42,44]. These discrepancies could reflect differences in species (rats versus mice), diets (control versus high fat versus high fructose) as well as disease models (aging versus obesity versus metabolic syndrome).

Aging increases risk of hyperglycemia and hyperinsulinemia, as they are signs of insulin resistance and glucose intolerance. Plasma glucose and insulin are commonly measured clinically but are less sensitive to detect impaired glucose homeostasis compared with insulin and glucose tolerance tests [40]. While our study showed age-related hyperinsulinemia, we did not observe evidence for hyperglycemia or effects of Ang-(1-7) to improve these measures. This is consistent with recent findings from our group and others showing that Ang-(1-7) does not impact fasting plasma insulin or glucose levels in obese mice [21,43,46]. Other studies support that Ang-(1-7) reduces fasting insulin but not glycemia in fructose-fed rats [20,44]. Longer-term treatment with Ang-(1-7) does improve both hyperinsulinemia and hyperglycemia in obese mice and fructose fed rats [44,47], suggesting improvements in insulin sensitivity precede the correction of hyperglycemia. It is possible, therefore, that longer durations of Ang-(1-7) treatment would correct hyperinsulinemia in aged mice but would be unlikely to impact glucose levels as aged mice were not hyperglycemic. 

Improvements in cardiovascular and metabolic function can be secondary to reductions in body mass, as weight loss independently decreases blood pressure, sympathetic activity, and insulin resistance [48,49]. Previous studies found that Ang-(1-7) decreases body mass and adiposity while increasing lean mass in obese male and female mice [42,43,50]. In the current study, Ang-(1-7) did not significantly decrease body mass or improve body composition in aged mice. It is possible again that longer treatment durations are needed to manifest changes in body composition in aged mice, given that energy balance is tightly regulated. Supporting this concept, previous studies showed body mass-independent improvements in cardiometabolic function in fructose-fed rats after four weeks of peripheral or central Ang-(1-7) treatment, whereas more chronic six-month treatment reduced body mass and adiposity in this model [44]. 

Overall, these studies showed that chronic Ang-(1-7) treatment improved integrated cardiometabolic function in aged mice by reducing blood pressure, insulin resistance, and cardiac sympathetic activity. The beneficial effects of Ang-(1-7) in aged mice occurred independent of changes in body mass or composition. Future studies will focus on circulating and tissue-specific mechanisms by which Ang-(1-7) ameliorates age-related cardiovascular and metabolic dysfunction, and potential sex differences. In particular, Ang-(1-7) could inhibit local or circulating components of Ang II pathways to elicit beneficial cardiometabolic effects. In support of this, Ang-(1-7) reduces plasma renin activity in fructose-fed rats [44], as well as ACE activity in canines and ex vivo in human cardiovascular tissues [51,52]. However, there is no information on the impact of Ang-(1-7) infusion on these RAS components during aging, and therefore, this remains an area of interest for future mechanistic research. 

## 4. Materials and Methods

### 4.1. Approvals

All procedures were approved by the College of Medicine Institutional Animal Care and Use Committee at the Pennsylvania State University (Approved 03/03/2016, ID 46841) and conformed to the NIH Guide for the Care and Use of Laboratory Animals. 

### 4.2. General Protocol

There were four groups of mice in this study: (1) young, saline infusion (*n* = 10); (2) young, Ang-(1-7) infusion (*n* = 10); (3) aged, saline infusion (*n* = 8); and (4) aged, Ang-(1-7) infusion (*n* = 10). Young adult (2-month-old) and aged (16-month-old) male C57BL/6J mice (Jackson Laboratory, Bar Harbor, ME, USA) were implanted with a subcutaneous osmotic mini-pump (Alzet 2006, Cupertino, CA, USA) to chronically deliver saline vehicle or Ang-(1-7) (400 ng/kg/min; Bachem, Torrance, CA, USA) for 6 weeks. This dose and administration route of Ang-(1-7) is known to improve cardiovascular and metabolic function in young adult mouse models of hypertension and obesity [12,21]. All mice were fed a standard chow diet (Teklad 2018, Envigo, Indianapolis, IN, USA) with ad libitum access to food and water. After 4 weeks of treatment, whole-body insulin action was assessed using standardized ITT and GTT, respectively. After 5 weeks of treatment, body composition and hemodynamics were measured. On the last day of treatment, mice were euthanized for the collection of blood and tissue samples. 

### 4.3. Insulin and Glucose Tolerance Testing

As recently described [43], for the ITT, four-hour fasted mice were injected intraperitoneally with insulin (0.75 U/kg, Novolin R), with glucose measured via a glucometer (Prodigy AutoCode, Charlotte, NC, USA) from tail vein blood samples at 15, 30, 60, 90, and 120 min post-injection. An additional blood sample was taken at baseline via a micro-hematocrit capillary tube to measure plasma insulin. For the GTT, overnight-fasted mice were injected intraperitoneally with 50% dextrose (2 g/kg), with glucose measured at baseline and at 15, 30, 60, 90, and 120 min post-injection. Plasma insulin was measured at baseline during the ITT as well as at baseline and 15 and 120 min post-injection during the GTT, using a mouse ultrasensitive insulin ELISA (Alpco Diagnostics, Salem, NH, USA). At least two days of recovery were allowed between ITT and GTT procedures. 

### 4.4. Body Composition

During the last week of treatment, body mass and composition were measured in conscious mice using a Bruker Minispec LF50 quantitative nuclear magnetic resonance analyzer (Billerica, MA, USA). Fat, lean, and fluid masses were reported as percentages of total body mass.

### 4.5. Blood Pressure and Heart Rate Measurements

Blood pressure and heart rate were measured in a subset of mice (*n* = 4–10/group) via an indwelling carotid artery catheter connected to a strain gauge-type transducer and a blood pressure analyzer (MP36R, Biopac Systems, Inc. Goleta, CA, USA), similar to our previous studies [12,21]. Blood pressure and heart rate were continuously recorded for at least 10 min, with average values during this period reported. Blood pressure and heart rate recordings were analyzed for indices of cardiac sympathetic and parasympathetic tone using frequency-domain spectral analysis methods (Acqknowledge 4 software, Biopac Systems, Inc.).

### 4.6. Euthanasia and Blood and Tissue Collection

Mice were euthanized via cardiac exsanguination under isoflurane anesthesia for the collection of blood and the isolation and weighing of heart and adipose (visceral epididymal, subcutaneous inguinal, and interscapular brown) tissues. 

### 4.7. Cardiac Gene Expression of RAS, Inflammatory and Sympathetic Markers

In a subset of aged mice treated with Ang-(1-7) versus saline (*n* = 4–6/group), the heart was flash frozen in liquid nitrogen and stored at −80 °C to examine RAS, sympathetic and inflammatory markers using quantitative real-time PCR. Frozen heart tissue (25 mg) was homogenized in QIAzol Lysis Reagent using a TissueLyser II, with total RNA extracted using RNAeasy Lipid Tissue Mini Kits and the automated processing QIAcube (Qiagen, Germantown, MD, USA). RNA concentration was measured with a NanoDrop spectrophotometer (ND-1000). cDNA was synthesized from total RNA using a high-capacity cDNA reverse transcription kit (ThermoFisher Scientific, Waltham, MA, USA). Quantitative real-time PCR was performed on a QuantStudio 12K Flex system (Applied Biosystems, Foster City, CA, USA) using mouse-specific Taqman gene primers (ThermoFisher Scientific). The primers used were: AT1R (*Agtr1a*, mm01957722_s1), Mas receptor (*Mas1*, mm00434823_s1), *ACE* (mm00802048_m1), *ACE2* (mm01159006 m1), *Th* (mm00447557_m1), TNFα (*Tnf*, mm00443258_m1), *IL6* (mm00446190_m1), and *IL10* (mm01288386_m1). Each sample was measured in triplicate with cycle threshold (CT) values normalized to the 18S ribosomal RNA (*Rn18s*; mm03928990_g1) housekeeping gene. Relative gene expression was determined using 2^−∆∆*C*t^ methods.

### 4.8. Statistical Considerations

Data are presented as mean ± SEM and analyzed by GraphPad Prism (Version 8.3.0, San Diego, CA, USA). Outcomes were compared among groups using two-way ANOVA to examine for main effects of age and drug as well as their interaction with a post-hoc Tukey test for multiple comparisons or between groups using an unpaired t-test. A *p*-value of <0.05 was considered statistically significant. 

## Figures and Tables

**Table 1:** Angiotensin (Ang)-(1-7) and body composition in aged mice.

Parameter, Unit	Young Saline	Young Ang-(1-7)	Aged Saline	Aged Ang-(1-7)	*P*_Age_	*P*_Drug_	*P*_Int_
*n*	10	10	8	10			
Body composition							
Body mass, g	30.0 ± 0.8	30.9 ± 0.6	46.1 ± 1.7 *†	44.4 ± 1.6 *†	0.001	0.740	0.302
Adiposity, %	7.0 ± 0.5	7.2 ± 0.7	14.6 ± 1.7 *†	16.7 ± 1.5 *†	0.001	0.328	0.451
Lean mass, %	69.7 ± 0.8	69.3 ± 0.9	60.7 ± 1.9 *†	58.2 ± 1.3 *†	0.001	0.251	0.403
Fluid mass, %	6.9 ± 0.1	6.7 ± 0.1	8.6 ± 0.2 *†	8.4 ± 0.1 *†	0.001	0.207	0.969
Adipose and heart tissue weights							
EPF, %	1.3 ± 0.2	1.6 ± 0.3	2.8 ± 0.5	3.7 ± 0.5 *†	0.001	0.152	0.457
SCF, %	1.0 ± 0.1	1.1 ± 0.2	2.3 ± 0.5	3.2 ± 0.4 *†	0.001	0.178	0.278
BAT, %	0.29 ± 0.05	0.29 ± 0.03	0.44 ± 0.06 †	0.45 ± 0.02 *†	0.001	0.809	0.877
Heart, %	0.36 ± 0.06	0.44 ± 0.01	0.48 ± 0.02	0.45 ± 0.03	0.038	0.362	0.085

Data are mean ± SEM and were analyzed by two-way ANOVA for main effects of age (*P*_Age_) and Ang-(1-7) versus saline treatment (*P*_Drug_) as well as their interaction (*P*_Int_) in young adult and aged mice. * *p* < 0.05 versus young saline and † *p* < 0.05 versus young Ang-(1-7) from post-hoc pairwise comparisons with Tukey correction for multiple comparisons. Body composition measurements and adipose and heart tissue weights are expressed as a percentage of total body mass. EPF, epididymal visceral white adipose tissue; SCF, inguinal subcutaneous white adipose tissue; BAT, interscapular brown adipose tissue.

## Abbreviations

ACE	angiotensin-converting enzyme
ACE2	angiotensin-converting enzyme 2
Ang	angiotensin
ARBs	angiotensin receptor blockers
AT1R	angiotensin II type 1 receptor
AUC	area under the curve
BAT	brown adipose tissue
EPF	epididymal visceral white adipose tissue
GTT	glucose tolerance test
IL6	interleukin 6
IL10	interleukin 10
ITT	insulin tolerance test
SCF	inguinal subcutaneous white adipose tissue
Th	tyrosine hydroxylase
TNFα	tumor necrosis factor α
